# Groundwater arsenic content in quaternary aquifers of the Red River delta, Vietnam, controlled by the hydrogeological processes

**DOI:** 10.1016/j.jhydrol.2022.127778

**Published:** 2022-06

**Authors:** Jolanta Kazmierczak, Trung Trang Dang, Rasmus Jakobsen, Hoan Van Hoang, Flemming Larsen, Helle Ugilt Sø, Nhan Quy Pham, Dieke Postma

**Affiliations:** a Geological Survey of Denmark and Greenland, Øster Voldgade 10, 1350 Copenhagen, Denmark; b Hanoi University of Science, Department of Geology, Hanoi, Vietnam; c Hanoi University of Mining and Geology, Department of Hydrology, Hanoi, Vietnam

**Keywords:** Groundwater arsenic, Quaternary aquifers, Numerical modeling, Sediment age, Flushed pore volumes, Arsenic transport

## Abstract

The relation between arsenic groundwater concentrations and hydrogeological processes was investigated in the proximal part of the Red River delta, Vietnam, west of the depression cone formed by the exploitation of groundwater in Hanoi. Flow paths in the Quaternary aquifers were modeled based on previously interpreted geological structure and hydrogeological data gathered during field work in 2014–2017. Sedimentary structures and simulated flow patterns were compared with the spatial distribution of the groundwater arsenic concentration.

The regression of the sea in the area started 4 ka BP in the Holocene. The low tectonic subsidence rate of the Red River delta led to intensive erosion and replacement of fine grained sediments of the sea level high stand by sandy channel belts, resulting in hydraulic connections between the Pleistocene and Holocene aquifers. The Pleistocene aquifer is recharged by both regional flow paths and naturally occurring vertical recharge through Holocene sand and clay layers. Young groundwater (<40 a) in the shallow Holocene aquifer generally discharges to surface water bodies. The shallow flow system is also seasonally recharged with surface water, as indicated by δ^18^O enrichment of groundwater and oscillating groundwater ages in wells in the vicinity of water channels. The deeper flow system discharges into the Red River and Day River or flows parallel to the rivers, toward the sea.

The overall pattern of arsenic groundwater concentrations (decreasing with increasing sediment age) is modified by groundwater flow. The arsenic contamination of the Pleistocene aquifer of the Red River delta is not only caused by the intensive groundwater abstraction in Hanoi, as reported previously, but also by the natural flow of high arsenic groundwater from Holocene to Pleistocene aquifers in areas located outside of the depression cone. Groundwater with < 50 µg L^-1^ arsenic is found in the Pleistocene aquifer close to the recharge zone in the mountains bordering the Red River delta and in the Holocene and Pleistocene aquifers where clay deposits were eroded. Close to the recent Red River channel, recharge of older Holocene and Pleistocene sediments occurs partially by arsenic-contaminated groundwater from the youngest Holocene aquifers, and here arsenic concentrations exceed 50 µg L^-1^. A high arsenic concentration is also present in the early Holocene-Pleistocene aquifer, beneath thick clay layers, indicating a limited extent of flushing and the inflow of fresh organic matter.

## Highlights

• High As levels in the Pleistocene aquifer imposed by natural hydrogeological processes.
• Preferential flow paths created due to the low subsidence rate in a river delta.
• Flow of high As groundwater from Holocene to Pleistocene aquifers.
• Surface derived organic matter seeping through thick, permeable clay layers.
• Groundwater As levels in Holocene aquifers related to the flushing extent.

## 1 Introduction

Natural contamination of groundwater by arsenic is a widespread problem in SE Asia (van Geen et al., 2003, Winkel et al., 2011, Kocar et al., 2014, Stahl et al., 2016, Gao et al., 2020). Groundwater arsenic concentrations in the floodplains of SE Asia are the result of interacting hydrochemical, sedimentary and geological processes. Arsenic is released to groundwater from iron hydroxides reduced by organic matter under anoxic conditions (Postma et al., 2007, Kocar et al., 2014, Postma et al., 2016, Gao et al., 2020). With time the reactivity of the organic matter in an aquifer decreases, and the rate of the reduction processes and the resulting groundwater arsenic content decreases (Lawson et al., 2016, Postma et al., 2016, Jakobsen et al., 2018), also due to large water volumes recharged through the aquifer, leaching sorbed arsenic from the sediment (Kocar et al., 2014, Jakobsen et al., 2018, Sø et al., 2018). Thus, groundwater arsenic levels decrease with increasing sediment age (Postma et al., 2012), but levels also depend on the sedimentary structure of the floodplain (Weinman et al., 2008, Donselaar et al., 2017). A highly heterogeneous distribution of arsenic in Holocene aquifers is often interpreted using flow (Michael and Voss, 2009, Hoque et al., 2017) or reactive transport models (Kocar et al., 2014, Jakobsen et al., 2018, Gao et al., 2020). However, three-dimensional (3D) models set up at the regional scale do not include detailed information regarding sedimentary structure and sediment age (Michael and Voss, 2009, Hoque et al., 2017). Furthermore, local, more detailed one-dimensional (1D) or two-dimensional (2D) models cannot capture regional flow patterns and the complexity of flow paths in meandering river environments (Kocar et al., 2014, Jakobsen et al., 2018, Gao et al., 2020). A regional 3D flow model including detailed information on sediment age and sedimentary structures could shed new light on the relation between sedimentary structure, sediment age, flow patterns and groundwater arsenic distribution at the regional scale.

Aquifers in older geological formations, including Pleistocene aquifers, usually have low arsenic content (van Geen et al., 2003, Hoque et al., 2017). This may be due to the decreased reactivity of organic matter with the sediment age (Postma et al., 2012, Lawson et al., 2016), oxic conditions in the sediments formed during the sea regression in Pleistocene (van Geen et al., 2003, van Geen et al., 2013, Stopelli et al., 2020) and a high number of recharged aquifer volumes (Jakobsen et al., 2018, Sø et al., 2018). The pattern is modified if an additional source of a fresh organic matter exists (Weinman et al., 2008, Nath et al., 2010, Lawson et al., 2016, Stahl et al., 2016) or if high arsenic groundwater recharges Pleistocene aquifers due to excessive groundwater abstraction (Winkel et al., 2011, Stahl et al., 2016, Donselaar et al., 2017, Stopelli et al., 2020). Excess groundwater abstraction may also result in transport of fresh organic matter into deeper aquifers and subsequent arsenic release (Harvey et al., 2002, Lawson et al., 2013) or land subsidence leading to clay layers shrinking and releasing arsenic contaminated groundwater from aquitards (Erban et al., 2013). Intensive groundwater abstraction below the city of Hanoi in the Red River delta, Vietnam, led to an intrusion of arsenic enriched groundwater from Holocene to Pleistocene aquifers (Winkel et al., 2011, van Geen et al., 2013) and intrusion of river water into Holocene aquifers and a subsequent arsenic release (Stahl et al., 2016, Stopelli et al., 2020). The contamination front in the Pleistocene aquifer is retarded by the oxidized Pleistocene sand and gravel (van Geen et al., 2013, Stopelli et al., 2020). Jakobsen et al. (2018) hypothesized, based on a 2D reactive transport model for the proximal part of the Red River delta outside the influence zone of the Hanoi groundwater abstraction, that arsenic is transported to the deeper aquifer along natural flow paths. This hypothesis is in line with the findings by Larsen et al. (2008) at the single meander scale, but has not been studied at the regional scale.

Clay layers are often reported as an important factor that prevents transport of arsenic from Holocene to Pleistocene aquifers in the floodplains of SE Asia due to transport retardation (van Geen et al., 2003). These clay layers act as natural barriers to redirect high arsenic groundwater toward discharge zones instead of to underlying aquifers (Hoque et al., 2017). Underlying Pleistocene aquifers are recharged by low arsenic groundwater of a regional flow system (Michael and Voss, 2009). However, arsenic levels in Pleistocene aquifers below thick clay layers in the Red River delta are high (Kazmierczak et al., 2022), and the related processes have not been previously investigated.

The objectives of this study were: (1) to evaluate and understand how the distribution of arsenic in Quaternary aquifer systems is related to the joint influence of sedimentary structure and hydrogeological processes, and (2) to investigate hydrogeological processes leading to the occurrence of high arsenic concentrations in the Pleistocene aquifer in the absence of pumping. For this purpose, a detailed model of the sedimentary architecture of the proximal part of the Red River delta (Kazmierczak et al., 2022) was developed into a three-dimensional flow model.

## 2 Study area

### 2.1 Geology of the Red River delta

**Fig. 1:**
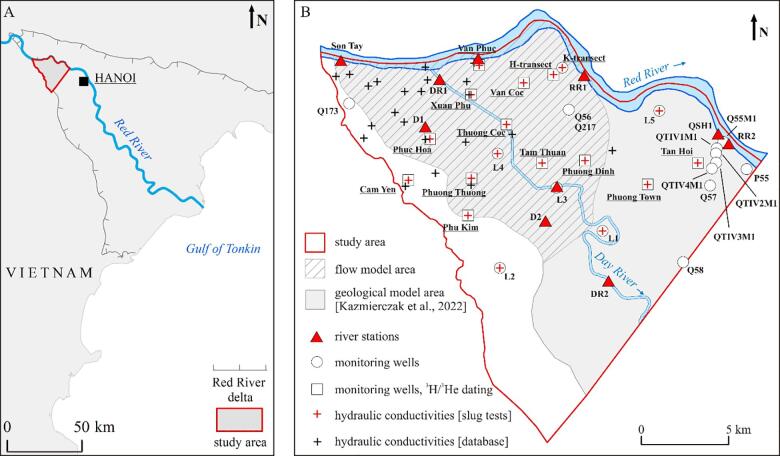
(A) Location of the Red River delta. (B) Field installations. Groundwater in transects and well pads with underlined names was sampled for stable isotopes analysis. (For interpretation of the references to color in this figure legend, the reader is referred to the web version of this article.)

**Fig. 2:**
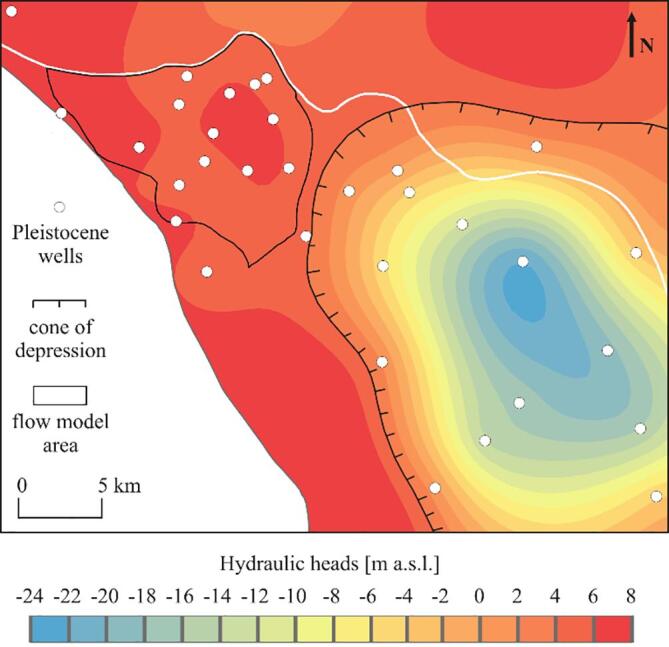
Extent of the Hanoi depression cone and the flow model boundaries with hydraulic heads distribution in the Pleistocene aquifer in the Red River delta in 2016. The flow model area corresponds to the dashed area in Fig. 1B. (For interpretation of the references to color in this figure legend, the reader is referred to the web version of this article.)

The research was conducted in the upper part of the Red River delta, Vietnam (Fig. 1A), west of the Hanoi depression cone (Fig. 2). The formation of the Red River delta started at approximately 9 ka before present (BP) during the post-glacial sea level transgression. Currently the delta covers a surface area of 10.3 × 10^3^ km^2^ (Tanabe et al., 2006).

The area was tectonically active until 5.5 Ma (Rangin et al., 1995). The current tectonic subsidence rate in the Red River delta is low, with a range of 0.04–0.12 mm a^-1^ (Mathers and Zalasiewicz, 1999). The bedrock consists of limestones, sandstones and siltstones displaced by numerous NW-SE striking faults, extending to the Gulf of Tonkin. The bedrock is overlain by a low permeable Neogene clay and a sequence of fining upward Pleistocene deposits: gravel of the Lechi and Hanoi formations and sand and clay of the Vinphuc formation. The Pleistocene deposits are followed by fine grained deltaic and floodplain deposits (Haihung formation). Sediments of the previous geological periods are cut through by fine to coarse grained sand of the Thaibinh formation deposited in the river channels with inserts of clay (Nghi et al., 1991). During the early Holocene (9–6 ka BP) a vertical accretion of the fine-grained deposits was the dominant sedimentary process. During sea level regression, which started at approximately 4 ka BP, the vertical accretion was exchanged with alluvial erosion and a lateral accretion of sand in the channel belts (Funabiki et al., 2012).

**Fig. 3:**
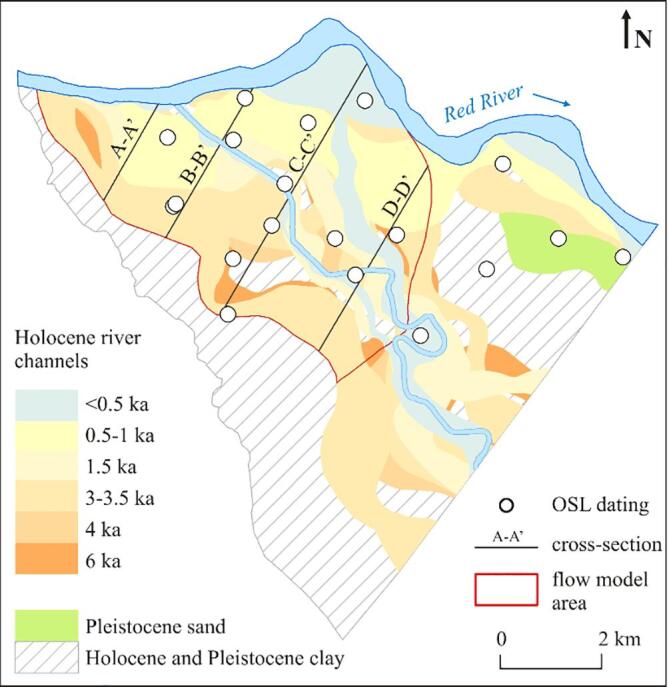
Geological model of the Red River floodplain based on Kazmierczak et al. (2022). Geological cross-sections are shown in Fig. 4. (For interpretation of the references to color in this figure legend, the reader is referred to the web version of this article.)

**Fig. 4:**
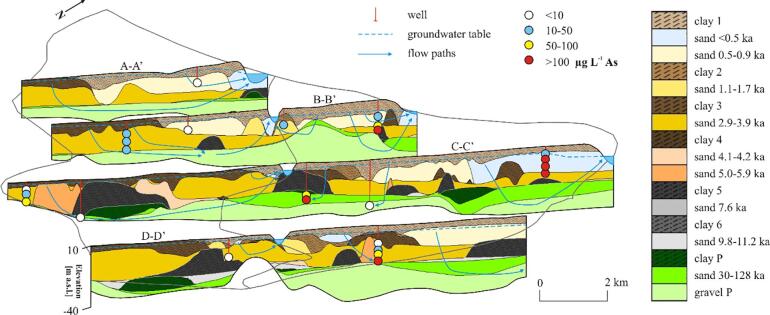
Hydrogeological cross-sections with modeled flow patterns and measured arsenic concentrations in the nearest wells. Geological model of the Red River floodplain developed based on Kazmierczak et al. (2022). (For interpretation of the references to color in this figure legend, the reader is referred to the web version of this article.)

The geological structure of the uppermost part of the Red River delta consists of a continuous layer of Pleistocene gravel overlain by islands of Pleistocene sand and clay. The Pleistocene deposits were covered by a thick layer of fine grained floodplain and deltaic deposits that were exposed to alluvial erosion at the SW margin of the floodplain and along the recent Red River course and replaced by coarse grained channel belt deposits and fine grained floodplain and oxbow lakes deposits (Fig. 3, Fig. 4; Kazmierczak et al., 2022).

Four hydrogeological units are distinguished in the Red River delta: lower and upper Pleistocene aquifers and lower and upper Holocene aquifers (Winkel et al., 2011).

### 2.2 Climate and hydrological conditions

**Fig. 5:**
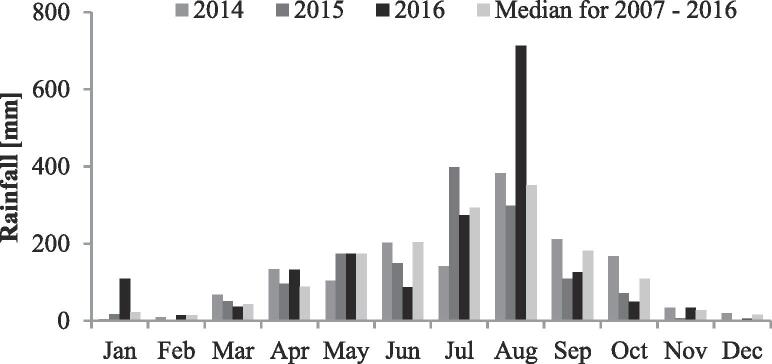
Precipitation at the meteorological station in Son Tay.

Precipitation, evaporation, river discharge and water levels in the western part of the Red River delta were monitored at the Son Tay climate station (Fig. 1B) by the Vietnam Meteorological and Hydrological Administration (vnmha.gov.vn). The climate in northern Vietnam is divided into a rainy season from May to October and a dry season from November to April. The rainy season accounts for about 70% of the total annual precipitation with a maximum rainfall of often over 300 mm in August. The annual rainfall at the Son Tay station reached 1483 mm in 2014, 1381 mm in 2015 and 1759 mm in 2016. Rainfall in January and August 2016 was well above the 2007–2016 median (Fig. 5). Evaporation measured in 2016 at the Son Tay station was 683 mm and the mean annual evaporation in the period 2007–2016 was 705 mm.

**Fig. 6:**
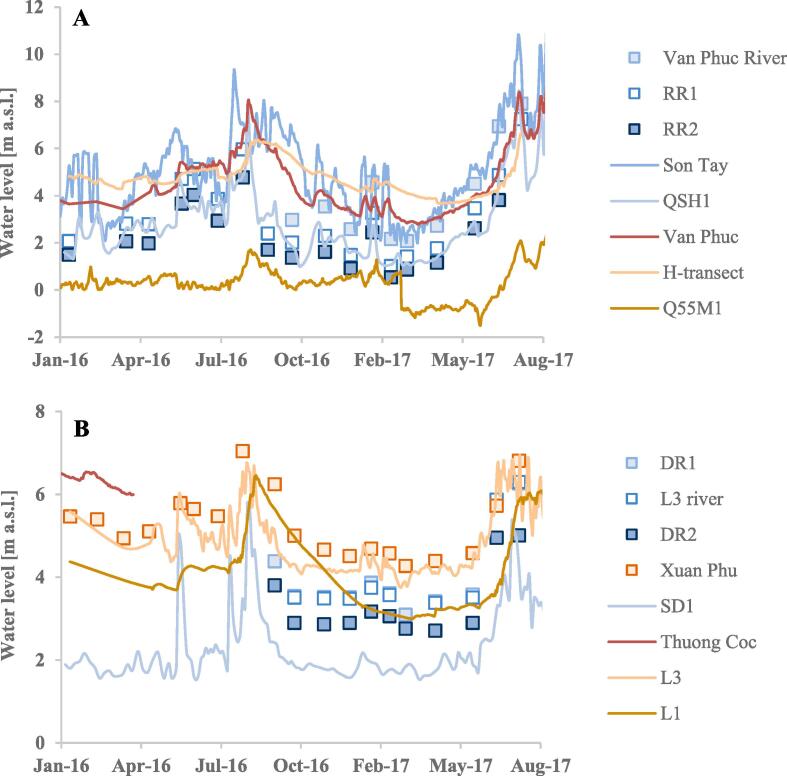
Water levels in the Red River (A) and the Day River (B) *vs*. groundwater levels in neighboring wells. (For interpretation of the references to color in this figure legend, the reader is referred to the web version of this article.)

The maximum water level of the Red River at the Son Tay station in 2014–2016 was 9.3 m a.s.l. and the minimum water level was 1.8 m a.s.l. (Fig. 6A). In the same period, the maximum and minimum discharge of the Red River was 7448 m^3^ s^−1^ and 557 m^3^ s^−1^, respectively (vnmha.gov.vn).

Interactions between the Red River and the Day River are heavily influenced by drainage systems and a dam. The dam in the Day River is located downstream from the L3 river station and was in the period 2014–2017 almost permanently closed. Two drainage systems in the modeled area open if the water level in the Red River exceeds 3.0 m in the dry season and 6.0 m in the rainy season (vnmha.gov.vn).

## 3 Methods

### 3.1 Hydrological conditions

The river stage of the Red River, Day River, and a discontinuous, larger river channel at the SW boundary of the research area was monitored at five, three and two river stations, respectively (Fig. 1B). The water level of the Red River at the meteorological Son Tay station was measured every six hours, and at the QSH1 station that belongs to the water resources monitoring network, every six days in the dry season and every three days in the rainy season. Monthly Red River water levels were monitored at three additional sites – Van Phuc, RR1 and RR2 from January 2016 to August 2017 (Fig. 1B).

Monthly water levels of the Day River were monitored from September 2016 to August 2017 at the two river stations (DR1 and L3), upstream of the dam in the Day River and one station (DR2) downstream of the dam (Fig. 1B). 21 km downstream of the research area there is a river station (SD1) of the water resources monitoring network where water level is measured every six days in the dry season and every three days in the rainy season.

The water levels at the two stations (D1 and D2, Fig. 1B) at the discontinuous river channel were measured twice, once in March and once in August 2017.

### 3.2 Well construction and hydrogeological conditions

12 multilevel well pads and two transects (white squares and K-transect in Fig. 1B), with one PVC well at each sampling depth down to the Pleistocene gravel and five single PVC wells (L1–L5) with screens at the bottom of the Holocene aquifer, were installed using water-jet drilling. The screens were 1 m long, had a diameter of 60 mm and were surrounded by a quartz sand pack. Each well was pumped clean directly after the installation and left to equilibrate for at least three months. In the research area there are also 11 wells of the national monitoring network (Fig. 1B).

Hydraulic heads were measured in all monitoring wells either manually or with a Solinst water level logger. The loggers were synchronized with the national monitoring network and set up to measure the hydraulic head every two hours. Hydraulic heads in the remaining wells were measured monthly. The hydraulic heads were monitored from December 2014 to August 2017, with the largest extent of the monitoring network in 2016. Water levels were corrected using the data from two barometric loggers installed at the Van Phuc and Phu Kim well pads located next to the Red River and close to the SW delta boundary, respectively (Fig. 1B).

Hydraulic conductivity of the aquifers was estimated via slug tests performed in wells of the 12 well pads and two transects and five single wells (Fig. 1B). Results of the slug tests conducted in the H- and K-transects were presented in Larsen et al. (2008). Slug tests in the remaining wells were performed in 2015. A slug test device was designed by a local manufacturer. It consisted of three solid aluminum cylinders, each one meter long with a diameter of 48 mm, sealed to avoid water intrusion. The test was performed by lowering the device below groundwater level (rising head test) and quickly removing the device from the water column (falling head test). Changes in the water pressure were measured automatically with the Solinst water level logger. Slug test results were interpreted in Aquifer Test version 3.0 using a Hvorslev analytical solution (Hvorslev, 1951).

At the well pads and transects a mean hydraulic conductivity of the Pleistocene and Holocene aquifers was calculated. An additional 28 hydraulic conductivities derived from single well pumping tests were extracted from the national monitoring database (Fig. 1B).

### 3.3 Groundwater chemistry

Groundwater samples were taken at least three months after the well installation using a Grundfos MP1 submersible pump. Electrical conductivity (EC), O_2_, pH, alkalinity and Fe(II) were measured in the field, and total As, As(III), Ca, Mg, Na, K, Mn, NH_4_^+^, Cl^-^, SO_4_^2-^, PO_4_^3-^, NO_3_^–^, CH_4_ and DOC in the laboratory. Sampling techniques and methodology used for the inorganic chemistry analysis are described in detail in Postma et al., 2007, Sø et al., 2018. Inorganic chemistry of groundwater in Holocene aquifers and hydrogeochemical processes relevant for arsenic release in the Red River delta were presented in studies by Postma et al., 2007, Postma et al., 2012, Postma et al., 2016 and Sø et al. (2018). In this study the total arsenic concentrations from both Holocene and Pleistocene aquifers were used. Additional four total arsenic concentrations measured in the tubewells by Winkel et al. (2011) were added to the analysis. Winkel et al. (2011) report an As measurement precision of ± 5%.

A boxplot of arsenic distribution in the sedimentary units derived by Kazmierczak et al. (2022) was produced. Median, 25th percentile, 75th percentile, minimum and maximum arsenic concentrations were calculated for four sedimentary units: (1) Holocene channel belt deposits with a burial age < 1.7 ka, (2) Holocene channel belt deposits with a burial age > 2.9 ka, (3) Pleistocene aquifer separated from Holocene aquifer by a thick (>10 m) clay layer, and (4) Pleistocene aquifer in the areas with hydraulic windows.

The groundwater age was determined using the ^3^H/^3^He method. Water samples for tritium were taken in 1 L PVC bottles and for helium and neon isotopes in 0.5 m long Cu tubes sealed at the ends with pinch-off clamps. The samples were analyzed at the University of Bremen (Sültenfuss et al., 2009). The age of the groundwater in the Holocene aquifers was presented in Sø et al. (2018). In this study additional data from the Pleistocene aquifers sampled at the Cam Yen and Tan Hoi well pads were used. Sø et al. (2018) presented a good agreement between the sum of ^3^H and ^3^He in groundwater of the Red River delta and the ^3^H time series in precipitation in Hong Kong. It indicates that there is no major mixing of groundwater with different ages and the uncertainty of the recharge age was estimated to be ± 2 a (Sø et al., 2018).

Water for the analysis of stable isotopes (δ^18^O and δD) was sampled in 20 mL PVC bottles. The isotopes were measured at the National Geological Survey of Denmark and Greenland (GEUS) using a Picarro L-2120i analyzer. The dataset was extended with the δ^18^O and δD concentrations in groundwater from the H-transect, K-transect, the Red River and precipitation in Hanoi presented in Larsen et al. (2008).

### 3.4 Flow model

**Fig. 7:**
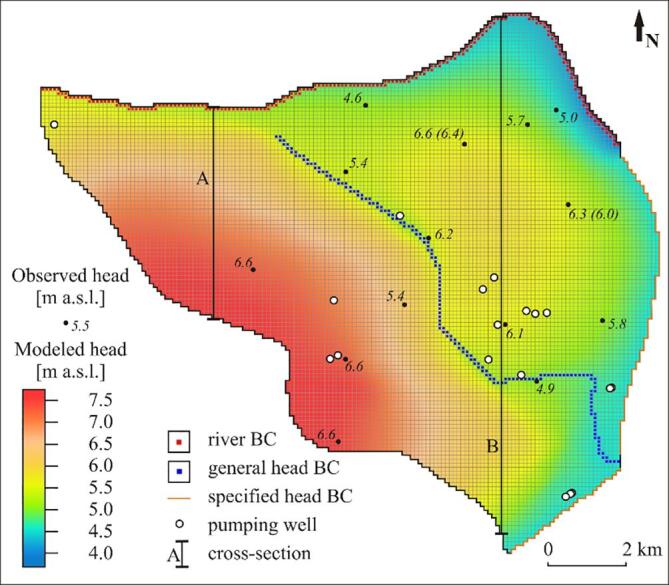
Steady state flow model setup with modeled *vs.* observed hydraulic heads. Numbers in brackets are hydraulic heads in the Pleistocene aquifer. Cross-sections A and B are shown in Fig. 8.

A 3D steady-state flow model for the proximal part of the Red River floodplain was set up using MODFLOW and the NWT solver (Harbaugh et al., 2000). The model had 38 numerical layers and a discretization of 100 m × 100 m in the XY directions (Fig. 7).

**Fig. 8:**
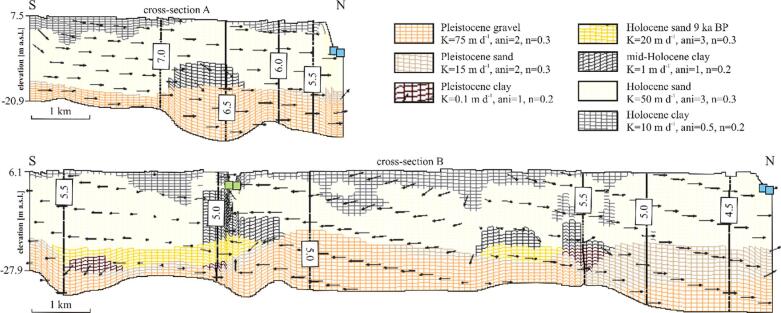
Modeled hydraulic heads (m a.s.l.) and flow paths (black arrows) in the western (upper cross-section) and eastern (lower cross-section) part of the study site. Blue squares indicate river boundary condition and green squares indicate general head boundary conditions. Calibrated hydraulic parameters for each geological unit are given. K is horizontal hydraulic conductivity, ani is vertical anisotropy and n is an effective porosity. Cross section locations are indicated in Fig. 7. (For interpretation of the references to color in this figure legend, the reader is referred to the web version of this article.)

Geology in the model domain was derived from the 3D sedimentary model (Kazmierczak et al., 2022; Figs. 1B, 3 and 4). The geological structure was simplified to seven units comprising: (1) Pleistocene gravel, (2) Pleistocene sand, (3) Pleistocene clay, (4) early Holocene sand deposited 9 ka BP, (5) thick, fine grained sediments of middle Holocene and early Holocene sand (6) and clay (7); Fig. 8.

The top of the model was interpolated to the Digital Elevation Model and bathymetry of the Red River measured by acoustic Doppler current profiler in seven transects perpendicular to the river shores in March 2000. A non-uniform recharge was assigned to the uppermost layer, decreasing with an increasing thickness of the uppermost clay layer. The recharge area included small water bodies that are abundant in the proximal part of the Red River delta. The bottom of the model corresponded to the bottom of the Pleistocene aquifer and was a no flow boundary.

The northern model boundary was set as a river boundary condition assigned to the cells of the uppermost layer (Fig. 7). The river conductance was 10 m^2^ d**^-1^** m**^−1^** and a mean water level in 2016 was assigned to the cells corresponding to the river stations. The Day River was separated from the Red River for most of 2016 and was discontinuous since the dam downstream from the L3 river station was closed. Thus, the Day River was represented using a general head boundary condition (Fig. 7), conductance of 100 m^2^ d**^-1^** and a water stage ranging from 4 to 3.8 m a.s.l. A specified head of 4.5 m a.s.l. was assigned to the eastern model boundary (Fig. 7). The boundary was set at the mean hydraulic head isoline bordering the extent of the Hanoi depression cone in 2016. The extent of the Hanoi depression cone was delineated using a mean hydraulic head in 2016 in 32 wells with screens in the Pleistocene aquifer (Fig. 2). Part of the wells were outside of the study area and belonged to the national monitoring network.

Local pumping wells in the study area, excluding single household tube wells, were inventoried by the National Center for Water Resources Planning and Investigation, Hanoi, Vietnam. In total 19 local pumping wells were mapped and, based on the data supplied by the owners, assigned to the grid with the middle of the screens from −0.6 to −35.5 m a.s.l. and a pumping rate from 1 to 700 m^3^ d**^-1^** (Fig. 7). A major part of the wells had the screen in the Pleistocene aquifer.

Groundwater age was simulated in MT3DMS (Zheng and Wang, 1999) with a zero-order production term of +1, following the approach by Engesgaard and Molson (1998). The production term was assigned to all active cells and represented the aging of groundwater with one day per day. Longitudinal and transverse dispersivities were set to zero and the recharge age was 0. The model was run for 2 ka in order to reach steady-state conditions.

Hydrogeological properties (hydraulic conductivity, anisotropy and porosity) of the geological units and the recharge rate were calibrated by trial and error method against the mean hydraulic heads in 2016 in 16 wells and 65 groundwater age measurements. Of these, eight wells were placed where Holocene and Pleistocene aquifers were connected, and eight in areas, where Pleistocene and Holocene aquifers were separated by a thick clay layer. Out of the latter, four wells had a screen in the Pleistocene aquifer and four in the Holocene aquifer. In the first step hydraulic conductivity, anisotropy and porosity were adjusted to minimize the Root Mean Squared Error (RMSE), and the solution with the lowest RMSE was selected for the groundwater age simulation. In the second step the porosity was adjusted to obtain the best fit between the measured and simulated groundwater age.

## 4 Results

### 4.1 Hydrogeological conditions

#### 4.1.1 Surface water

Water levels in the rivers fluctuated from a minimum in the dry season to a maximum in the rainy season (Fig. 6). The amplitude of fluctuations was 6 m in the Red River and 3 m in the Day River. The response of water levels in the Red River to changes in rainfall magnitude is immediate, while the highest fluctuations of water stage in the Day River were related to discharge directed from the Red River to the Day River.

The mean annual river stage of the Red River in 2016 was 5.5 m a.s.l. at the upstream Son Tay river station and 2.4 m a.s.l. at the downstream RR2 river station. The mean annual hydraulic gradient between the stations was 0.0001. Mean water levels in the Day River from September to December 2016 at the DR1 and L3 river stations located upstream from the dam were nearly equal (4.7 m a.s.l., Fig. 6B). This indicates a hydraulic gradient near zero in the Day River upstream from the dam. The mean water level of 4.7 m a.s.l. was also found at the DR2 river station, downstream from the dam. The mean annual river stage in 2016 at the SD1 river station, located approximately 20 km downstream from the DR2 river station, was 2.2 m a.s.l. The mean annual hydraulic gradient in the Day River downstream from the dam was 5 × 10^-5^. The water level at stations D1 and D2 at the SW discontinuous river channel (Fig. 1B) was above the groundwater level in the nearest wells during both dry and rainy season.

**Fig. 9:**
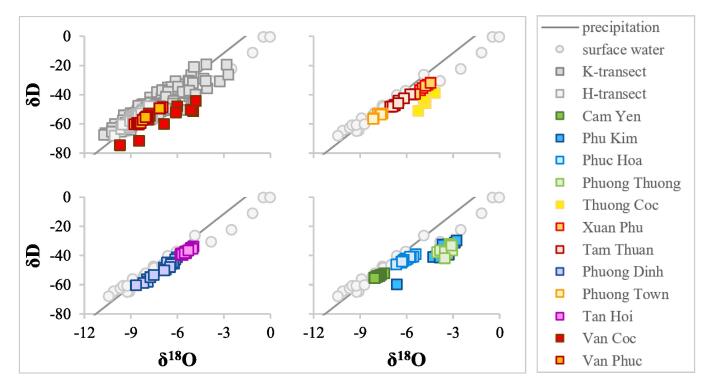
Distribution of stable isotopes in groundwater (squares) compared to the Hanoi precipitation curve and surface water in the Red River. Precipitation, surface water, H-transect and K-transect data sets are from Larsen et al. (2008). (For interpretation of the references to color in this figure legend, the reader is referred to the web version of this article.)

The stable isotope composition of the Red River water during 2008 ranged from –10.4 to –0.03 for δ^18^O and from –68.0 to –0.3 for δD (Fig. 9; Larsen et al., 2008).

#### 4.1.2 Groundwater

The Pleistocene and Holocene aquifers of the study area were mostly hydraulically connected, and only locally separated by Pleistocene and early to mid Holocene clay layers (Fig. 3, Fig. 4). The clay layers were more abundant in the SE part of the study area (Fig. 4). The hydraulic heads in the Holocene aquifer decreased towards the Red River and the Day River and were highest in the NE part of the research area, where a thick clay layer occurred, and at the SW delta boundary (Fig. 7). During 2016, the mean hydraulic heads in the Holocene aquifer not affected by the Hanoi groundwater abstraction varied from 2.6 m a.s.l. at well L5 near the Red River in the NE part of the study area to 6.9 m a.s.l. at well QTIV3M1, located SE from well L5. Hydraulic heads in the Holocene aquifer, connected to the Pleistocene aquifer and influenced by the groundwater abstraction were below 0 m a.s.l., and a minimum mean head in 2016 equaled −0.1 m a.s.l. at well Q55M1, located near the Red River in the NE corner of the study area (Fig. 6). Hydraulic heads in the Pleistocene aquifer decreased rapidly towards the east from an isoline of 4.5 m a.s.l. (a 2016 mean) that enclosed the Hanoi depression cone (Fig. 2). Mean hydraulic heads in 2016 in the Pleistocene aquifer varied from −5.4 m a.s.l. at well Q58, at the eastern border of the study area, to 6.9 m a.s.l. at well Q173, located approximately 20 km NW from well Q58. A downward vertical gradient was observed between Holocene and Pleistocene aquifers both in areas influenced by groundwater pumping and outside of the depression cone (Fig. 7).

Hydraulic heads, similar to river stages, fluctuated from a minimum in the dry season to a maximum in the rainy season (Fig. 6). The highest annual groundwater level fluctuations were in the well pads located close to the Red River, e.g. 6.5 m at Van Phuc. Annual hydraulic head fluctuations decreased with increasing distance from the Red River, e.g. from 6.5 m at Van Phuc to 3 m at the H-transect well pads (for location see Fig. 1B). The lowest annual groundwater level fluctuations were in the well pads located>2 km from the Red River and ranged from 0.7 to 3.9 m with the highest fluctuations (3.2–3.9 m) along the Day River and its tributaries. Extreme rainfall events had an influence only on hydraulic heads in the well pads located near the Red River or the Day River.

In 2016–2017, the hydraulic heads in the wells located close to the Red River and the Day River were higher than the river water level for most of the year (Fig. 6). Reversals of the hydraulic gradient occurred during the rainy season, e.g. Van Phuc and H-transect well pads located close to the Red River and a single well L3 located near the Day River (Fig. 6). Well Q55M1, located near the Red River in the NE corner of the study area, was influenced by groundwater abstraction and its hydraulic head was below the river stage throughout the entire period. The mean hydraulic head gradient at a distance>2 km from the Red River and the Day River was 0.0002.

Stable isotope values in groundwater varied from −4.4 to −8.8 for δ^18^O and from −31.9 to −60.4 for δD (Fig. 9). Some of groundwaters in the well pads were enriched in δ^18^O and δD (e.g. K-transect, Thuong Coc, and Xuan Phu in Fig. 9). Stable isotopes ratios in the remaining samples were close to the precipitation in Hanoi (Larsen et al., 2008). Groundwater with the lowest content of δ^18^O and δD was found in Van Phuc and H-transect in the northern part of the study area, Cam Yen located on a the Pleistocene terrace along the SW model boundary, and at Phuong Town with wells screened in the early Holocene deposits covered by a thick clay layer (Fig. 9).

**Fig. 10:**
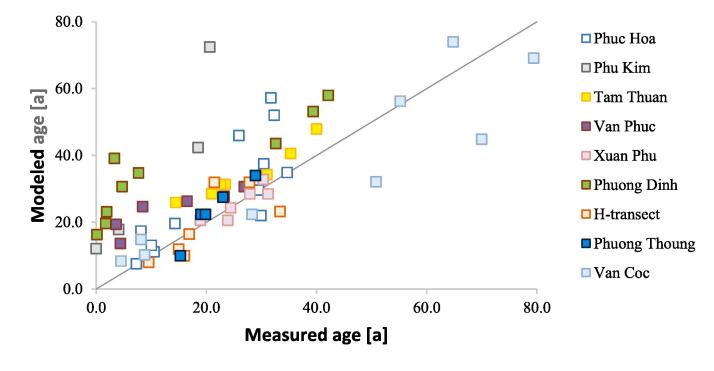
Measured *vs.* modeled groundwater age.

The measured groundwater age in general increased linearly with depth (Sø et al., 2018) and ranged from 0.1 a in the Holocene aquifer at Phuong Dinh, located approximately 1 km from the Day River and SE boundary of the flow model, to 79.4 a in the Pleistocene aquifer at Van Coc located in the northern part of the study area, approximately 1 km from the Red River (Fig. 10). In some of the vertical profiles near surface water bodies the groundwater age oscillated in the shallow part of the aquifer (Sø et al., 2018).

#### 4.1.3 Aquifer properties

Hydraulic conductivity in the aquifers measured in this study (section 3.3) and by Larsen et al. (2008) and derived from the national monitoring database varied from 1 to 240 m d**^-1^** and was highest in the Pleistocene aquifer consisting of gravel and coarse sand. The mean hydraulic conductivity equaled 36 m d**^-1^** in Holocene sand, 12 m d**^-1^** in Pleistocene sand and 105 m d**^-1^** in Pleistocene gravel. The standard deviation in hydraulic conductivity was 26, 12 and 74 m d**^-1^** in Holocene sand, Pleistocene sand, and Pleistocene gravel, respectively. The Holocene sand deposited in channel belts had a rather homogenous hydraulic conductivity at each well pad/transect and varied by an order of magnitude. The mean hydraulic conductivity of the Holocene aquifer at well pad/transects varied from 9 m d**^-1^** at Phu Kim to 93 m d**^-1^** at Phuc Hoa, both located in SW old river channel (Fig. 1B).

### 4.2 Flow model

#### 4.2.1 Model calibration

The calibrated hydraulic conductivity varied from 0.1 m d**^-1^** in Pleistocene clay to 75 m d**^-1^** in Pleistocene gravel (Fig. 8). The calibrated vertical anisotropy was 0.5 to 1 in clay layers, and 2 to 3 in Pleistocene and Holocene sand and gravel (Fig. 8). Calibrated effective porosity ranged from 0.2 in clay layers to 0.3 in sand and gravel. The assigned recharge ranged from 2.5 × 10^-4^ to 5.5 × 10^-4^ m d**^-1^**, decreasing with an increasing thickness of the top clay layer and was lowest in some of the areas along the SE flow model boundary. RMSE for hydraulic heads of the final model was 0.7 m. The absolute value of the residual head ranged from 0.04 to 1.1 m. The lowest deviations between measured and modeled hydraulic heads were along the Red River and the Day River (Fig. 4). The simulated groundwater age exceeded the measured age in part of the samples at especially Phuong Dinh located near the recent Day River channel, and Phu Kim at the SW flow model boundary but otherwise, with some scatter, were comparable to the observations (Fig. 10).

#### 4.2.2 Flow patterns and velocities

The highest hydraulic heads were modeled along the SW model boundary and the lowest along the Red River and the Day River courses and at the eastern model boundary (Fig. 7, Fig. 8). Higher heads were also predicted in the NE area of the model. Simulated heads ranged from 3.9 to 7.6 m a.s.l. and showed a slight downward gradient.

**Fig. 11:**
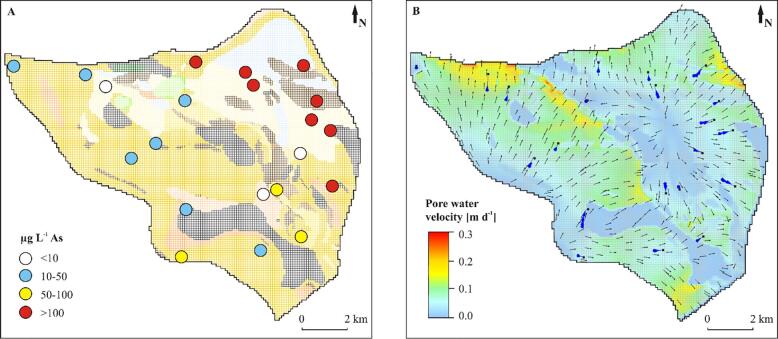
Interpreted geological structure and arsenic distribution (A) and modeled flow patterns and flow velocities (B) in layer 14 cutting through the middle part of the Holocene aquifer at *ca.* −5 m a.s.l. Blue arrows in panel B indicate flow paths to the sampled wells and a travel time equal to 10 a. Colors of the geological units correspond to Fig. 4. (For interpretation of the references to color in this figure legend, the reader is referred to the web version of this article.)

**Fig. 12:**
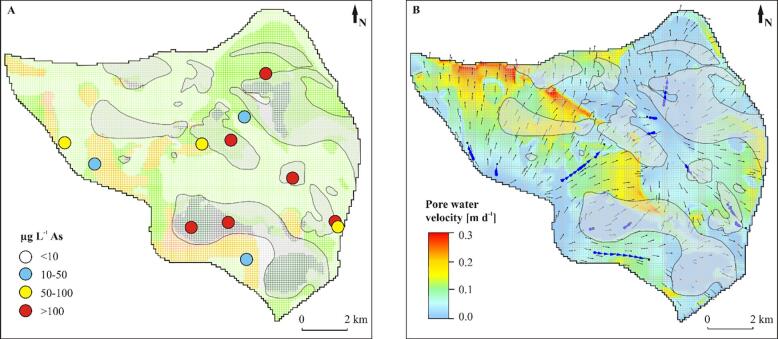
Interpreted geological structure and arsenic distribution (A) and modeled flow patterns and flow velocities (B) in layer 30 cutting through the early Holocene and Pleistocene aquifers at *ca.* −25 m a.s.l. Blue arrows in panel B indicate flow paths to the sampled wells and a travel time equal to 10 a. Greyed areas indicate places of connection between late and mid-Holocene aquifers and early Holocene and Pleistocene aquifers. Colors of the geological units correspond to Fig. 4. (For interpretation of the references to color in this figure legend, the reader is referred to the web version of this article.)

The Red River and the Day River were major discharge paths of groundwater and drained 43 × 10^3^ m^3^ d**^-1^**, which was 80% of the total recharge. 18% of groundwater flowed through the old, abandoned channel belts, parallel to the recent Red River and Day River courses and discharged at the eastern model boundary (Fig. 8, Fig. 11, Fig. 12). The remaining 2% of the recharge was abstracted by the pumping wells.

Groundwater flow was from south to north at the western part of the study area and changed into a more complex flow system in the eastern part, where recharged groundwater created a radial diverging flow toward the Red River, the Day River and the eastern model boundary (Fig. 8, Fig. 11, Fig. 12). Groundwater flow was nearly vertical through the uppermost clay layers and shallow Holocene aquifer and changed into horizontal flow in the deeper part of the Holocene aquifer and in the Pleistocene aquifer (Fig. 4, Fig. 11, Fig. 12). Part of the groundwater from the Holocene aquifer recharged the Pleistocene aquifer either via hydraulic windows or through the clay islands (Fig. 4, Fig. 8, Fig. 11, Fig. 12). The travel time to the wells with screens in the Holocene aquifer was a few decades while in the Pleistocene aquifer simulated travel times reached 100 a (Fig. 11, Fig. 12).

**Fig. 13:**
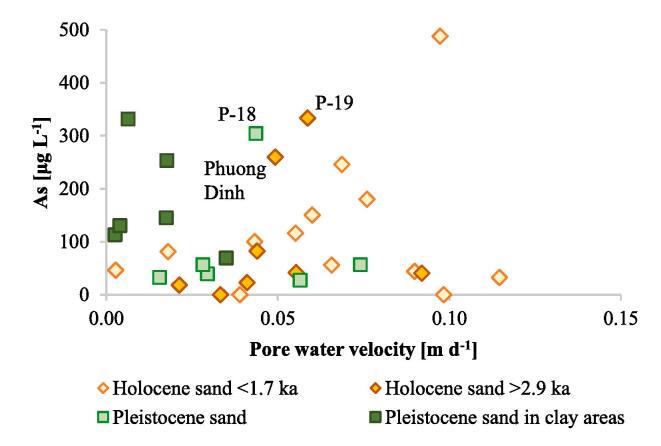
Groundwater arsenic concentration *vs.* pore water velocities and sediment age. Flow paths recharging wells P-18, P-19 and Phuong Dinh originate from Holocene channel belts with a burial age < 1.7 ka.

The range of simulated flow velocities in Holocene and Pleistocene aquifers was similar (Fig. 13). Flow velocities were highest along rivers and in the Pleistocene aquifer (Fig. 11, Fig. 12). The lowest simulated flow velocities in the Pleistocene aquifer were below thick clay layers (Fig. 12, Fig. 13). The flow velocities were up to 0.3 m d**^-1^** (Fig. 13) and near 10^-3^ m d**^-1^** in clays and 10^-2^ m d**^-1^** in sands.

### 4.3 Arsenic distribution

The highest groundwater arsenic concentrations in the Holocene aquifer were found in the NE part of the study area (Fig. 4, Fig. 11) while in the Pleistocene aquifer high groundwater arsenic concentrations were spread throughout a larger area (Fig. 4, Fig. 12).

**Fig. 14:**
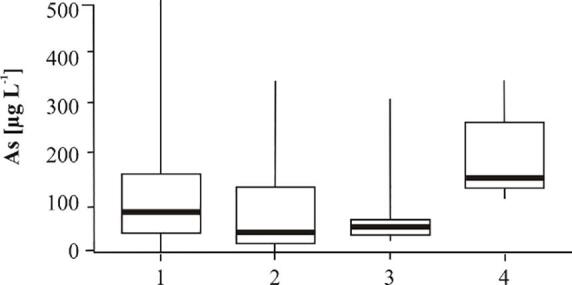
A boxplot of arsenic distribution in geomorphological structures of the Red River delta: (1) Holocene channel belt deposits with a burial age < 1.7 ka, (2) Holocene channel belt deposits with a burial age > 2.9 ka, (3) Pleistocene aquifer in areas of hydraulic windows, and (4) Pleistocene aquifer separated from Holocene aquifer by a thick (>10 m) clay layer.

In the Holocene aquifer the highest arsenic concentrations are present in the recent Red River channel belt with a sediment burial age < 1 ka BP and in the older Holocene channel belts overlain by younger deposits of the Day River (Fig. 4, Fig. 11). Median and maximum arsenic concentrations in channel belts with a burial age < 1.7 ka were 81 and 488 μg L^-1^, respectively (Fig. 14). The groundwater arsenic concentrations are lower (median concentration of 41 μg L^-1^) along the SW model boundary, where sand was buried at > 2.9 ka BP (Fig. 3, Fig. 4, Fig. 11, Fig. 14). The lowest groundwater arsenic concentrations are at the western part of the study area in both early and late Holocene deposits. Here Pleistocene and mid-Holocene clay layers were eroded, and the flow velocities are high (Fig. 4, Fig. 11, Fig. 12, Fig. 13). Groundwater arsenic concentrations were also low (<10 μg L^-1^) in the uppermost 1–2 m of Holocene aquifer, increasing with depth up to 488 μg L^-1^ (light blue deposits < 0.5 ka in Fig. 4).

All wells with screens in the Pleistocene aquifer in the modeled area had groundwater that exceeded the WHO guideline for arsenic content in drinking water (>10 µg L**^-1^**), with minimum arsenic concentration of 27 μg L^-1^ below the hydraulic windows and 113 μg L^-1^ below thick clay layers (Fig. 12, Fig. 13, Fig. 14). The highest groundwater arsenic concentrations in the Pleistocene aquifer were found below thick Pleistocene and mid-Holocene clay layers (median of 145 μg L^-1^) and downstream from the recent (burial age < 1 ka BP) channel belt deposits (Fig. 12, Fig. 14). Groundwater arsenic concentrations exceeding 10 µg L**^-1^** As were also found downstream from the channel belts buried at > 2.9 ka BP and in the areas of the higher flow velocities (Fig. 4, Fig. 12).

## 5 Discussion

### 5.1 Hydrogeological conditions

#### 5.1.1 Groundwater-surface water interactions

The rivers in the Red River delta are the major groundwater discharge zones (Fig. 8, Fig. 11, Fig. 12). River water only recharges the aquifers during the rainy season. A hydraulic gradient reversal as indicated by measured water levels (Fig. 6) is confirmed by δ^18^O and δD values in groundwater near the surface water bodies being similar to surface water values (Larsen et al., 2008) showing an enrichment in the heavy isotopes compared to the precipitation curve (Fig. 9). Vertical profiles of groundwater age also indicate flow reversals with plumes of younger groundwater in the uppermost part of the profiles (Sø et al., 2018).

The study of groundwater-surface water interactions would require a transient flow model in order to simulate hydraulic gradient reversals. However, in this study a steady-state flow model describing mean annual conditions was set up. The reason being that the focus of this study was on flow velocities, water volumes flushed through the aquifers and the main flow paths in Holocene and Pleistocene aquifers. Detailed, local short-term groundwater-surface water interactions were beyond the study scope.

Seasonal changes of recharge and river stages only cause fluctuations in local, near surface flow paths (Michael and Voss, 2009). In the proximal part of the Red River delta the seasonal hydraulic head fluctuations decrease from 6 to 1 m with an increasing distance from the Red River and the Day River. Flow reversals only occur along the Red River, while Day River is recharged by groundwater most of the year (Fig. 6). In the transient flow model set up for the H-transect area by Larsen et al. (2008), the hydraulic gradient reversals occur within a maximum distance of 1.5 km from the Red River. Furthermore, the recharge of an aquifer with river water in the rainy season corresponds to only 5% of the groundwater recharge to the river in the dry season (Larsen et al., 2008), and low hydraulic gradients in the aquifers (Fig. 8) prevent fast inland migration of the river water. Thus, flow patterns are only disturbed near the Red River, and flow reversals do not have a significant impact on the arsenic mass balance in the studied aquifers (Larsen et al., 2008). Arsenic release in the Red River delta takes place in the anoxic part of aquifers (Postma et al., 2007; 2016) that have thickness on the order of tens of meters. Therefore, immobilisation of arsenic in the uppermost 1–2 m of the aquifer due to oxygen inflow during seasonal water level decrease (Larsen et al., 2008) would also have a minor influence on the observed distribution of arsenic concentrations at the regional scale. The limited extent in time and space of flow reversals, the small volume of aquifer influenced by arsenic immobilisation due to oxic conditions, and the focus on regional flow paths (Fig. 7) all support the choice of a steady-state flow model.

#### 5.1.2 Calibrated parameters

The model was calibrated against mean hydraulic heads and river water levels in 2016, when a higher than mean rainfall for the period 2007–2016 took place in January and August (Fig. 5). However, the extreme rainfall events did not influence the mean Red River stage and groundwater levels (Fig. 6). The Red River stage in the rainy season in 2016 was up to 10 m a.s.l. and did not exceed a mean water stage of nearly 12 m a.s.l. in the previous years (Larsen et al., 2008).

Calibrated hydrogeological properties in the proximal part of the Red River delta (Fig. 8) compare well with parameters used in previous modeling studies by Larsen et al., 2008, Jusseret et al., 2009. Hydraulic conductivities of Holocene sand deposited in channel belts, early Holocene sand of the delta initiation phase and Pleistocene gravel were 50, 20 and 75 m d**^-1^**, respectively. Hydraulic conductivities in the model set up for the Hanoi area (Jusseret et al., 2009) had the same order of magnitude and equaled 43 m d**^-1^** in Holocene channel belt sand and 78 m d**^-1^** in Pleistocene gravel. Mean hydraulic conductivity in Holocene sand in the H-transect area was 30 m d**^-1^** (Larsen et al., 2008). Calibrated hydraulic conductivities also fit into the ranges of measured hydraulic conductivities that were 9 to 93 m d**^-1^** in Holocene sand and 105 m d**^-1^** (standard deviation of 74 m d**^-1^**) in Pleistocene gravel.

Calibrated hydraulic conductivities for clay and silt are up to three orders of magnitude higher than in the models set up by Jusseret et al., 2009, Thu and Fredlund, 2000 for the Hanoi area. These models, however, assumed no recharge via the clay layers. The difference in hydraulic conductivities decreases to one order of magnitude for a scenario with aquifer recharge through both sand and clay layers (Jusseret et al., 2009). Higher hydraulic conductivities in clay layers in the uppermost part of the Red River delta compared to the Hanoi area may be a result of erosion processes. The uppermost clay layers in the modeled area are weathered and intensively fractured and thus highly permeable (Larsen et al., 2008, Jakobsen et al., 2018). Highly permeable clay windows responsible for rapid recharge of aquifers were also described in the Mekong delta, Cambodia (Lawson et al., 2016). Higher permeability of clay in the study area results in low arsenic concentrations in groundwater below the thin uppermost clay layers (Fig. 4), indicating an inflow of oxic groundwater that prevents the reduction of iron hydroxides (Larsen et al., 2008). Nearly linearly increasing groundwater age with depth in the Holocene aquifer also indicates recharge through overlying clay layers (Sø et al., 2018). Flow paths with a significant vertical component in the Holocene aquifer were also simulated in the 3D flow model (dark blue arrows in Fig. 11) and in 2D by Jakobsen et al. (2018).

Alluvial deposits in the Red River delta are well sorted (Larsen et al., 2008). It is visible in the nearly homogeneous vertical profiles of measured hydraulic conductivity and in the gamma-log profiles showing a uniform natural gamma radiation (Kazmierczak et al., 2022). Thus, a low vertical anisotropy was set in the model area with a range from 1 to 3. Porosity assigned to the model ranged from 0.2 to 0.3 and is within a range 0.001 to 0.3 used in the flow model for the Hanoi area (Jusseret et al., 2009). A mean porosity of Holocene sand estimated in the local study at the H-transect was 0.39 (Larsen et al., 2008). Calibrated recharge rates on the order of 10^-4^ m d**^-1^**, which compares well with the recharge of 3 × 10^-4^ m d**^-1^** in the local model for the H-transect (Larsen et al., 2008).

The developed model represents well the distribution of hydraulic heads and groundwater age in the proximal part of the Red River delta, and hydraulic properties of the aquifers are within the ranges reported in previous studies (Larsen et al., 2008, Jusseret et al., 2009). However, as a trial and error calibration method was applied, it must be noted that the developed model is perhaps one of many plausible solutions, but not one that is objectively determined to be the best given the available data.

### 5.2 Processes controlling groundwater arsenic distribution

#### 5.2.1 Sedimentary structure *vs.* Arsenic distribution

The arsenic distribution in the Holocene aquifer in the Red River delta resembles the heterogeneous pattern observed in Bangladesh (van Geen et al., 2003, Weinman et al., 2008) and Cambodia (Kocar et al., 2014). Arsenic in the aquifers of the Red River delta is released from iron oxyhydroxides reduced by organic matter under anoxic conditions (Postma et al., 2007, Postma et al., 2016). The reactivity of organic matter deposited in the alluvial sediments, and therefore the reduction rate of iron hydroxides and mobilization of arsenic, decreases with time (Lawson et al., 2016, Postma et al., 2016). Furthermore, the arsenic concentration decreases with increasing number of pore volumes flushed through the aquifer (Postma et al., 2012, Jakobsen et al., 2018, Sø et al., 2018). Thus, assuming homogeneous hydrogeological conditions, the groundwater arsenic content decreases with increasing sediment age (Postma et al., 2012).

In the shallow Holocene aquifer, a clear link exists between sediment age and the groundwater arsenic level, although exemptions were also observed (Fig. 4, Fig. 11, Fig. 13, Fig. 14). The highest groundwater arsenic levels (median of 81 μg L**^-1^**) are found in the youngest Holocene sediments buried < 1.7 ka BP (Fig. 14). These deposits are distributed along recent courses of the Red River and the Day River (light blue and light yellow color in Fig. 4, Fig. 11A). Groundwater with lower arsenic levels (median of 41 μg L**^-1^**) occurs in the SW part of the model area that consists of channel belt deposits with a burial age > 2.9 ka BP (Fig. 4, Fig. 11A, and 14).

The relation between groundwater arsenic content and sediment age is not present in the deeper (and older) parts of the Holocene aquifer where high arsenic concentrations can be found. There are also parts of the Pleistocene aquifer, which is normally low in arsenic, due to a different geological history, where high arsenic levels occur (cross-sections C–C’ and D–D’ in Fig. 4, Fig. 12, Fig. 13, Fig. 14). The highest groundwater arsenic concentrations in the Pleistocene aquifer are below clay layers with a thickness > 10 m (greyed areas in Fig. 12, Fig. 13, Fig. 14). High arsenic levels below or adjacent to thick clay layers are common in Holocene aquifers (Weinman et al., 2008, Nath et al., 2010, Kocar et al., 2014, Donselaar et al., 2017). Clay layers overlying Pleistocene aquifers were so far reported as a factor causing retardation or even preventing arsenic transport to the Pleistocene aquifers (Hoque et al., 2017). However, clay layers in the proximal part of the Red River delta lead to increased arsenic contamination of the Pleistocene aquifer (Fig. 12).

High groundwater arsenic levels in the older Holocene river channels and Pleistocene deposits (Fig. 13, Fig. 14) are related to a limited extent of aquifer flushing (Section 5.2.2; Jakobsen et al., 2018, Sø et al., 2018), and migration of arsenic from shallow to deeper aquifers (Section 5.2.3; Winkel et al., 2011). Transport of organic matter from e.g. clay layers or surface water bodies into the deeper aquifers may be an additional factor that increases reduction rates of iron hydroxides and subsequent arsenic release (Postma et al., 2007, Weinman et al., 2008, Kocar et al., 2014, Lawson et al., 2016, Stahl et al., 2016).

#### 5.2.2 Number of flushed pore volumes *vs.* Arsenic distribution

A relation between groundwater arsenic content and the number of recharged aquifer volumes has been documented for the Holocene aquifers (Fig. 11, Fig. 12, Fig. 13), as previously reported by Jakobsen et al., 2018, Sø et al., 2018. A similar effect would be expected for the Pleistocene aquifers, but due to the more complex history of the Pleistocene aquifers they cannot be compared directly with the Holocene aquifers. The number of recharged pore volumes is an effect of the recharge rate, changes in flow patterns with shifting river courses and the sediment burial age (Jakobsen et al., 2018, Sø et al., 2018). The modeled range of pore water velocities is similar in all aquifers of the Red River floodplain (Fig. 13), however, the time available for the aquifer flushing after the sediment burial varies from 128 ± 11 ka to 0.46 ± 30 ka (Kazmierczak et al., 2022). The lowest groundwater arsenic levels are found in the western part of the study site where clay layers were eroded and replaced with coarse grained channel belt deposits at 2.9–5.9 ka BP (Fig. 4, Fig. 11, Fig. 12). Here the high number of flushed pore water volumes, resulting from the increased pore water velocities and the longer time available for aquifer flushing, removed arsenic contamination from early Holocene aquifers and presumably also the Pleistocene aquifer (Fig. 13, Fig. 14). The highest groundwater arsenic levels in the Holocene aquifer are in the NE part of the study site comprised of channel belt deposits with a burial age < 1.7 ka (Fig. 4, Fig. 12), despite relatively high pore water velocities (Fig. 13). According to the model study by Postma et al. (2016) the groundwater arsenic level in the proximal part of the Red River floodplain reaches a maximum during the first 1200 a. Thus, high arsenic levels in the youngest channel belts are related to high rates of arsenic mobilisation and limited extent of flushing.

High arsenic concentrations are also found in Pleistocene aquifers below thick clay layers (greyed areas in Fig. 12). Recharge of the Pleistocene aquifer through thick clay layers (>10 m) in the study area is lower than through the outcrops of the Holocene or Pleistocene aquifers (Fig. 13). The occurrence of thick clay layers limits recharge of the aquifer and thus pore water velocity and thereby the ability to flush groundwater high in released arsenic (Fig. 12, Fig. 13). High groundwater arsenic levels in Pleistocene aquifers may additionally be a result of clay layers acting as a source of organic matter (Weinman et al., 2008, Kocar et al., 2014, Lawson et al., 2013, Lawson et al., 2016).

The relation between the extent of flushing and groundwater arsenic levels in Holocene aquifers in the proximal part of the Red River delta was described by Sø et al. (2018). Sø et al. (2018) estimated, based on the sediment burial age, aquifer depth and the vertical flux rate calculated from ^3^H/^3^He profiles, that the groundwater arsenic concentration decreases < 10 μg L**^-1^** after approximately 200 pore volumes were flushed through the aquifer. However, extremely high arsenic concentrations were also found in older Holocene aquifers with pore water velocities of approximately 0.5 m d^-1^ (Fig. 13). The 2D conceptual, transient reactive transport model for arsenic in the Red River aquifers set up by Jakobsen et al. (2018) showed that high arsenic concentrations in the older aquifers may be related to the downward transport of contamination. Furthermore, high arsenic groundwater accumulates in stagnation zones below the major river channels and the flushing process is delayed until the river channel shifts position and changes the flow pattern (Jakobsen et al., 2018).

#### 5.2.3 Arsenic distribution modified by flow patterns

The Red River delta developed under low tectonic subsidence rates (Mathers and Zalasiewicz, 1999) that led to erosion of older deposits during sea level regression at 4 ka BP. It resulted in connections between the Pleistocene and Holocene aquifers and transport of high arsenic groundwater from the youngest alluvial channels to older Holocene and Pleistocene aquifers (e.g. wells Phuong Dinh, P-18 and P-19 in Fig. 11, Fig. 12, Fig. 13). This is contrary to the thick deposits in the Bengal Basin with a high tectonic subsidence rate, where Holocene and Pleistocene aquifers are well separated, and hierarchical flow systems are imposed by the many interleaved discontinuous clay layers (Hoque et al., 2017). Here the Pleistocene aquifers are less contaminated with arsenic, unless excess groundwater pumping takes place in the deeper aquifers (Hoque et al., 2017). Additionally, the high anisotropy in aquifers of the Bengal Basin imposes a regional flow system in the Pleistocene aquifer and prevents seepage of arsenic contaminated groundwater from the youngest Holocene deposits (Michael and Voss, 2009). At the proximal part of the Red River delta, despite the complex geological structure, the Pleistocene aquifer has both, regional and local flow systems (Fig. 4, Fig. 8). The flow paths of the latter transports arsenic to the Pleistocene aquifer, as also suggested by Jakobsen et al. (2018).

All groundwater arsenic concentrations in the Pleistocene aquifer in the study area exceed WHO guideline of 10 µg L**^-1^** (Fig. 12). It appears contrary to the occurrence of oxic sediments in the Pleistocene aquifer and potentially high number of recharged aquifer volumes (Sø et al., 2018). Groundwater arsenic concentrations in the Pleistocene in areas where thick clay layers have been eroded (wells outside of the greyed areas in Fig. 12) are similar to those in Holocene aquifers with burial age > 2.9 ka (Fig. 13, Fig. 14). It indicates that groundwater from older Holocene aquifers with > 10 µg L**^-1^** arsenic (Fig. 11) flows vertically down into the Pleistocene aquifer, which is supported by modeled flow paths to the wells along the SW model boundary screened in the Pleistocene aquifer (Fig. 12). The vertical flow is facilitated by low sediment anisotropy within the channel belt deposits. Transport of the groundwater enriched in arsenic from Holocene to Pleistocene aquifers in the proximal part of the Red River delta was reported at the single meander scale by Larsen et al. (2008). Anomalous arsenic concentrations in deeper groundwater were also found in the Bengal Basin where hydraulic windows existed (Hoque et al., 2017).

Increased groundwater arsenic concentrations in Pleistocene aquifers are a result of complex flow and transport patterns imposed by heterogeneities in the sedimentary structure of the floodplain and topographical differences also observed in Cambodia (Kocar et al., 2014), Bangladesh and Vietnam (Jakobsen et al., 2018). The capacity of the oxic sandy Pleistocene sediments to retard arsenic contamination, described for a locality east of Hanoi (van Geen et al., 2013, Stopelli et al., 2020) and from Bangladesh (van Geen et al., 2003), is apparently much lower in the studied area, probably due to the coarse grained nature of the sediment.

Annual hydraulic head fluctuations enable the intrusion of oxygen with recharging groundwater to the uppermost part of Holocene aquifer where clay layers are thin (Larsen et al., 2008). Infiltrated oxygen oxidizes organic matter and prevents reduction of iron hydroxides and a subsequent arsenic release (Larsen et al., 2008, Kocar et al., 2014). Furthermore, it leads to precipitation of secondary iron hydroxides and re-adsorption of the released arsenic (Larsen et al., 2008). Thus, arsenic concentrations in the Holocene aquifer near the groundwater table are < 10 μg L^-1^ and are limited to the uppermost 2 m of Holocene aquifer (Fig. 4).

### 5.3 Implications for drinking water resources

Elevated arsenic concentrations in Pleistocene aquifers of the Red River delta were previously reported (Winkel et al., 2011, Stahl et al., 2016) and interpreted as a result of excessive groundwater abstraction. However, our study site is located outside of the Hanoi depression cone (Fig. 2). The local wells in the area only abstract 2% of the total aquifer recharge, and hydraulic heads are not lower in the vicinity of the local abstraction wells compared to the surrounding areas (see Phuong Thuong and Tam Thuan well pads in Fig. 7). Furthermore, hydraulic head measurements in the vertical profiles and model results both indicated a small downward hydraulic gradient between the Holocene and Pleistocene aquifer (Fig. 7). Under these conditions, in a highly conductive Pleistocene aquifer with regional flow paths, low groundwater arsenic concentrations are expected, especially beneath the thick clay layers (Michael and Voss, 2009, Hoque et al., 2017). However, high arsenic levels in the Pleistocene aquifer of our study site are found in both areas with and without local abstraction wells (Fig. 4, Fig. 12). Elevated arsenic levels in the Pleistocene aquifer of the uppermost part of the Red River delta are therefore not an outcome of excess groundwater abstraction, as reported in the Hanoi area (Winkel et al., 2011). Instead, they are a result of hydrogeological processes, such as a limited extent of flushing and transport of contamination from shallow to deeper aquifers (Section 5.2). Remediation of drinking water resources to limit arsenic contents in the proximal part of the Red River delta is therefore not possible, e.g. by change of pumping rates, due to the natural origin of the contamination. However, additional deterioration of groundwater quality in the aquifers outside of the expanding Hanoi depression cone (Fig. 7; Bui et al., 2012) can be prevented by sustainable water resources management.

## 6 Conclusions

Groundwater enriched in arsenic was found in Pleistocene aquifers of the Red River delta outside of the Hanoi depression cone. High groundwater arsenic levels in Pleistocene aquifers of the Red River delta may be the result of a limited extent of flushing and inflow of arsenic contaminated groundwater from Holocene aquifers along natural flow paths, and not only, as previously described, inflow induced by excess groundwater abstraction.

Due to the low tectonic subsidence rate in the Red River delta, clay layers separating aquifers have been extensively removed by erosion. As a result, arsenic enriched groundwater from Holocene aquifers is transported naturally through hydrogeological windows into the Pleistocene aquifers, which are often by default considered as a source of low arsenic water.

In addition, thick clay layers, if permeable, may provide organic matter to underlying aquifers. This may induce iron hydroxides reduction and arsenic release not only in younger Holocene aquifers, but also in Pleistocene aquifers, resulting in high groundwater arsenic levels.

## CRediT authorship contribution statement

**Jolanta Kazmierczak:** Conceptualization, Methodology, Formal analysis, Investigation, Resources, Writing – original draft, Visualization, Supervision, Project administration. **Trung Trang Dang:** Formal analysis, Investigation, Data curation, Writing – original draft, Visualization. **Rasmus Jakobsen:** Methodology, Investigation, Writing – review & editing. **Hoan Van Hoang:** Investigation, Resources, Data curation. **Flemming Larsen:** Writing – review & editing, Supervision. **Helle Ugilt Sø:** Formal analysis, Writing – review & editing. **Nhan Quy Pham:** Resources, Data curation. **Dieke Postma:** Conceptualization, Resources, Writing – review & editing, Supervision, Funding acquisition.

## Declaration of Competing Interest

The authors declare that they have no known competing financial interests or personal relationships that could have appeared to influence the work reported in this paper.

## References

[b0005] Bui D.D., Kawamura A., Tong T.N., Amaguchi H., Nakagawa N. (2012). Spatio-temporal analysis of recent groundwater-level trends in the Red River Delta, Vietnam. Hydrogeol. J..

[b0010] Donselaar M.E., Bhatt A.G., Ghosh A.K. (2017). On the relation between fluvio-deltaic flood basin geomorphology and the wide-spread occurrence of arsenic pollution in shallow aquifers. Sci. Total Environ..

[b0015] Engesgaard P., Molson J. (1998). Direct simulation of groundwater age in the Rabis Creek Aquifer Denmark. Groundwater.

[b0020] Erban L.E., Gorelick S.M., Zebker H.A., Fendorf S. (2013). Release of arsenic to deep groundwater in the Mekong Delta, Vietnam, linked to pumping-induced land subsidence. PNAS.

[b0025] Funabiki A., Saito Y., Phai V.V., Hieu N., Haruyama S. (2012). Natural levees and human settlement in the Song Hong (Red River) delta, northern Vietnam. Holocene.

[b0030] Gao Z.P., Jia Y.F., Guo H.M., Zhang D., Zhao B. (2020). Quantifying geochemical processes of arsenic mobility in groundwater from an inland basin using a reactive transport model. Water Resour. Res..

[b0035] Harbaugh, A.W., Banta, E.R., Hill, M.C., McDonald, M.G., 2000. MODFLOW-2000, the U.S. Geological Survey modular ground-water model. User guide to modularization concepts and the ground-water flow process. Open File Rep. 00-92, USGS, Denver, CO, pp. 121. doi:10.3133/ofr200092.

[b0040] Harvey C.L., Swartz C.H., Badruzzaman A.B.M., Blute N.K., Winston Y., Ali A., Jay J., Beckie R., Niedan V., Brabander D., Gates P.M., Ashfaque K.N., Islam S., Hemond H.F., Ahmed M.F. (2002). Arsenic Mobility and Groundwater Extraction in Bangladesh. Sci..

[b0045] Hoque M.A., Burgess W.G., Ahmed K.M. (2017). Integration of aquifer geology, groundwater flow and arsenic distribution in deltaic aquifers – a unifying concept. Hydrol. Process..

[b0050] Hvorslev, M.J., 1951. Time Lag and Soil Permeability in Ground-Water Observations, Bull. No. 36, Waterways Exper. Sta. Corps of Engrs, U.S. Army, Vicksburg, Mississippi, pp. 1-50.

[b0055] Jakobsen R., Kazmierczak J., Sø H.U., Postma D. (2018). Spatial variability of groundwater arsenic concentration as controlled by hydrogeology: conceptual analysis using 2-D reactive transport modeling. Water Resour. Res..

[b0060] Jusseret S., Tam V.T., Dassargues A. (2009). Groundwater flow modelling in the central zone of Hanoi, Vietnam. Hydrogeol. J..

[b0065] Kazmierczak J., Postma D., Dang T., Hoang V.H., Larsen F., Hass A.E., Hoffmann A.H., Fensholt R., Pham N.Q., Jakobsen R. (2022). Groundwater arsenic relation to sedimentology and stratigraphy in the Red River delta. Vietnam. *Sci. Total Environ.*.

[b0070] Kocar B.D., Benner S.G., Fendorf S. (2014). Deciphering and predicting spatial and temporal concentrations of arsenic within the Mekong Delta aquifer. Environ. Chem..

[b0075] Larsen F., Pham N.Q., Dang N.D., Postma D., Jessen S., Pham V.H., Nguyen T.B., Trieu H.D., Tran L.T., Nguyen H., Chambon J., Nguyen H.V., Ha D.H., Huen N.T., Duc M.T., Refsgaard J.C. (2008). Controlling geological and hydrogeological processes in an arsenic contaminated aquifer on the Red River flood plain. Vietnam. *Appl. Geochem.*.

[b0080] Lawson M., Polya D.A., Boyce A.J., Bryant C., Mondal D., Shantz A., Ballentine C.J. (2013). Pond-Derived Organic Carbon Driving Changes in Arsenic Hazard Found in Asian Groundwaters. Environ. Sci. Technol..

[b0085] Lawson M., Polya D.A., Boyce A.J., Bryant C., Ballentine C.J. (2016). Tracing organic matter composition and distribution and its role on arsenic release in shallow Cambodian groundwaters. Geochim. Cosmochim. Acta.

[b0090] Mathers S., Zalasiewicz J. (1999). Holocene sedimentary architecture of the Red River Delta. Vietnam. *J. Coastal Res.*.

[b0095] Michael H.A., Voss C.I. (2009). Controls on groundwater flow in the Bengal Basin of India and Bangladesh: regional modeling analysis. Hydrogeol. J..

[b0100] Nath B., Mallik S.B., Stüben D., Chatterjee D., Charlet L. (2010). Electrical resistivity investigation of the arsenic affected alluvial aquifers in West Bengal, India: usefulness in identifying the areas of low and high groundwater arsenic. Environ. Earth Sci..

[b0105] Nghi T., Ngo Quang Toan, Do Thi Van Thanh, Nguyen Dinh Minh, Nguyen Van Vuong (1991). Quaternary sedimentation of the principal deltas of Vietnam. J. SE Asian Earth Sci..

[b0110] Postma D., Larsen F., Nguyen T.M.H., Mai T.D., Pham H.V., Pham Q.N., Jessen S. (2007). Arsenic in groundwater of the Red River floodplain, Vietnam: controlling geochemical processes and reactive transport modeling. Geochim. Cosmochim. Acta.

[b0115] Postma D., Larsen F., Nguyen T.T., Pham T.K.T., Jakobsen R., Pham Q.N., Tran V.L., Pham H.V., Murray A.S. (2012). Groundwater arsenic concentrations in Vietnam controlled by sediment age. Nature Geosci..

[b0120] Postma D., Pham T.K.T., Sø H.U., Hoang V.H., Vi M.L., Nguyen T.T., Larsen F., Pham H.V., Jakobsen R. (2016). A model for the evolution in water chemistry of an arsenic contaminated aquifer over the last 6000 years, Red River floodplain. Vietnam. *Geochim. Cosmochim. Acta*.

[b0125] Rangin C., Klein M., Roques D., Pichon X.L., Trong L.V. (1995). The Red River fault system in the Tonkin Gulf. Vietnam. *Tectonophysics*.

[b0130] Sø H.U., Postma D., Lan V.M., Trang T.K., Kazmierczak J., Nga D.V., Pi K., Koch C.B., Viet P.H., Jakobsen R. (2018). Arsenic in Holocene aquifers of the Red River floodplain, Vietnam: Effects of sediment-water interactions, sediment burial age and groundwater residence time. Geochim. Cosmochim. Acta.

[b0135] Stahl M.O., Harvey C.F., van Geen A., Sun J., Trang P.T.K., Lan V.L., Phuong T.M., Viet P.H., Bostick B.C. (2016). River bank geomorphology controls groundwater arsenic concentrations in aquifer adjacent to the Red River, Hanoi Vietnam. Water. Resour. Res..

[b0140] Stopelli E., Vu T.D., Tran T.M., Pham T.K.T., Pham H.V., Lightfoot A., Kipfer R., Schneider M., Eiche E., Kontny A., Neumann T., Glodowska M., Patzner M., Kappler A., Kleindienst S., Rathi B., Cirpka O., Bostick B., Prommer H., Winkel L.H.E., Berg M. (2020). Spatial and temporal evolution of groundwater arsenic contamination in the Red River delta, Vietnam: Interplay of mobilisation and retardation processes. Sci. Total Environ..

[b0145] Sültenfuss J., Roether W., Rhein M. (2009). The Bremen mass spectrometric facility for the measurement of helium isotopes, neon and tritium in water. Isot. Environ. Health Stud..

[b0150] Tanabe S., Saito Y., Vu Q.L., Hanebuth T.J.J., Ngo Q.L., Kitamura A. (2006). Holocene evolution of the Song Hong (Red River) delta system, northern Vietnam. Sed. Geol..

[b0155] Thu T.M., Fredlund D.G. (2000). Modelling subsidence in the Hanoi City area. Vietnam. *Can. Geotech. J.*.

[b0160] van Geen A., Zheng Y., Versteeg R., Stute M., Horneman A., Dhar R., Steckler M., Gelman A., Small C., Ahsan H., Graziano J.H., Hussain I., Ahmed K.M. (2003). Spatial variability of arsenic in 6000 tube wells in a 25 km^2^ area of Bangladesh. Water Resour. Res..

[b0165] van Geen A., Bostick B.C., Trang P.T.K., Lan V.M., Mai N.N., Manh P.D., Viet P.H., Radloff K., Aziz Z., Mey J.L., Stahl M.O., Harvey C.F., Oates P., Weinman B., Stengel C., Frei F., Kipfer R., Berg M. (2013). Retardation of arsenic transport through a Pleistocene aquifer. Nature.

[b0170] vnmha.gov.vn Vietnam Meteorological and Hydrological Association. Accessed on 07.03.2022.

[b0175] Weinman B., Goodbred S.L., Zheng Y., Aziz Z., Steckler M., van Geen A., Singhvi A.K., Nagar Y.C. (2008). Contributions of floodplain stratigraphy and evolution to the spatial patterns of groundwater arsenic in Araihazar. Bangladesh. *Geol. Soc. Am. Bull.*.

[b0180] Winkel L.H.E., Pham T.K.T., Vi M.L., Stengel C., Amini M., Nguyen T.H., Pham H.V., Berg M. (2011). Arsenic pollution of groundwater in Vietnam exacerbated by deep aquifer exploitation for more than a century. P. Natl. Acad. Sci. USA (PNAS).

[b0185] Zheng C., Wang P.P. (1999). MT3DMS: A modular three-dimensional multispecies transport model for simulation of advection, dispersion, and chemical reactions of contaminants in groundwater system.

